# Supplementary material for: Isolation, Characterization, Genome Analysis and Host Resistance Development of Two Novel Lastavirus Phages Active against Pandrug-Resistant Klebsiella pneumoniae
Source: Viruses. 2023 Feb 25;15(3):628. doi: 10.3390/v15030628 (PMC10052179; doi:10.3390/v15030628)
Supplement: Supplementary file 1 [file viruses-15-00628-s001.zip › viruses-2141529-supplementary.pdf]

Supplementary material

1. Supplementary tables

Table S1: Rho-factor-independent terminators in genomes of LASTA/SJM3

Sr. No	Position of transcription terminator	Strand	Sequence	e-value
1	52	-	ACCAGCCACTAGCCCCTGGTTTCAGCCGGGGGCTTTTTATTTTTTG	-16.80
2	3693	+	TTCTGCTCTTGCGTTGCCGGGCCAGCAACGTTTTCGGCCTTC	-7.10
3	5787	+	TCTGACCAGTACAGGGAATTATCCTTGTTTTCGCTGTCC	-6.30
4	12183	-	TATGGTTTCTCCCGGCGTTGGTACTGGCGCTGGTTTTGCTACATC	-11.50
5	13550	+	GGGGATCATTACCGGTTTGCCGGTTTTGGTGTCGT	-5.60
6	16509	-	GAAAATTGCGCGCTGTCACCCTCTGGGACGGCTTTTCAGAGTGT	-7.90
7	16691	-	TCAGTAAACTTGGTAATGGTCGTGCGTTGCCcTTTTGTCTATCA	-8.10
8	21912	-	GTTATGATTAAGCCCCGAAAGGGGCTTTTTCGTATGC	-11.00
9	23680	-	ATAAAAACACGGTTGGACGGCATGTTCAGCTTTTCGCTGTGG	-6.70
10	26320	-	CAGCATGGGAGGTTGGCGGCAGCACAAGCCAGCTTTTCAACTTTT	-8.70
11	30200	+	TGACCGTCGTCGGCAGCATCTTCAGCGCTGTCTTCGTCTTCGCC	-8.10
12	30707	-	AATCACCAGCAGGGGCGAAAGCCCCTGTCTTTTCATC	-11.00
13	32413	+	GCAAACACTCATGTCATGCCAATGGCGTTTTTGCTTTGA	-4.60
14	33139	+	CCCGCGCTCTTTGTTCAGCTCGCGCAGCTGGGCATTATATTCGCGG	-9.50
15	33657	+	TCCTGCATAGCCTGCTGCTCGGCAGCGGcTTGCTGTTGCTG	-9.80
16	36968	+	CACGGTGGTAATGCGCTTCTCCTTGCGCATTTTGACCATCT	-8.00
17	38042	-	CACATGCCTCACCGCATTATAGCGGTTTTCTGTGCAA	-6.30
18	40700	-	GGTGGTTTTAATGTCGCCCTGTTCCTGGCGGCGTTTGTTGTAGCT	-9.90
19	40878	-	TAAAACGAAACAGGAGCGCCTATGAGCGCTCCTTTCTTTTGGTGTC	-14.80
20	40880	+	CACCAAAAGAAAGGAGCGCTCATAGGCGCTCCTgTTTCGTTTTATGG	-15.30
21	42085	-	TGCGGGAAATCCCGCCCCGGCCGCTGGTGGTTTTAACGTGCC	-5.90

22	43130	+	CCGCTCTACGGCGGCGATCCATTGCTGTTTTGATTATTC	-5.20
23	47378	+	CGGGGTAAGACTCGGAAGAGCGAAATGGCTTTCGATTTTGCGATATG	-5.70
24	50336	-	TGAGCATTGGAGGCCAACCAGGCCTTTTCCTCGTCG	-6.80
25	53425	-	TAACGCGAGGCTGGCGAGCGCCGCCGTTTTTGCTGGTG	-6.60
26	55853	+	AGTCGGATGGTGGGGAAGTTCACGCTTCCCCcTTTTCAATCTGT	-13.10
27	56531	-	GCCTTATCCTTGTCACCGCACGGTGGCTCTGTCTTCGAC	-8.70
28	59000	-	CGATTTGGCGACGCGCTTCAGCGCGTTTTTTATCTGT	-8.10
29	59527	+	CATGGCTTTTTCCTCGGTTGGTTGTCGAGGTTATTTTCCTCA	-6.30
30	59620	-	GATAAATGTTCTGACCGCCAGGGTCGcTTTTTCAACAAG	-6.20
31	60485	-	TCGACTTCCATGCCCGCCGTACCAGCGGGCTTTTTTTTGTTC	-12.00
32	61208	+	ATCTGCGGCCTTCGTAGCTGCTGCTGCGGccTTTTGCGCTGCT	-9.60
33	61368	-	GTTCAGCATTGCCGGTGCTTGCGCCGGTTTTGCCGCGAG	-9.50
34	61726	-	TGCAGCCATTACCGCTTTTATCGGCGGaTTTTTTTGGCTC	-7.00
35	62196	+	ATCGATGCCTTTGGCGATGAGCCGTTTTTTGAAGAT	-4.80

Table S2: Putative conserved regulatory elements of LASTA/SJM3 genome as predicted by PHIRE

Sr. No	End Position	Start position	Sequence
1	14595	14576	CTGCTGGTCCTGCTGCTGCT
2	08714	08695	CTACTGTTCCTGCGGCTGGT
3	13005	12986	TGGCTGGTCCTGCTGCTGGG
4	14598	14579	CTGGTCCTGCTGCTGCTGCT
5	32834	32815	CTGCTGCTGCTTCGGCTGCT
6	32837	32818	CTGCTGCTTCGGCTGCTGCT
7	32852	32833	CTGCTGGGCCGCCTGCTCCT
8	33332	33313	CTGCTGGGCTTTCTGCTGCT
9	33344	33325	CTGCTGCTCCTGTTGGTCCT
10	33353	33334	CTGTTGGTCCTGCTGCTCTT
11	33362	33343	CTGCTGCTCTTCCTGCTTCT
12	33365	33346	CTGCTCTTCCTGCTTCTGTT
13	33624	33605	CTGCTGTTCCTGCTGTTGCT
14	33660	33641	CTGCTGGGCCTGTTGCTCCT
15	58923	58942	CTGGTGGTGCTGTTGCTGGT (consensus)
